# Water Stress Scatters Nitrogen Dilution Curves in Wheat

**DOI:** 10.3389/fpls.2018.00406

**Published:** 2018-04-06

**Authors:** Marianne Hoogmoed, Victor O. Sadras

**Affiliations:** South Australian Research and Development Institute, Adelaide, SA, Australia

**Keywords:** water stress, carbon isotope discrimination, phenology, *Triticum aestivum*, water-soluble carbohydrates

## Abstract

Nitrogen dilution curves relate a crop’s critical nitrogen concentration (%N_c_) to biomass (W) according to the allometric model %N_c_ = *a* W*^-b^*. This model has a strong theoretical foundation, and parameters *a* and *b* show little variation for well-watered crops. Here we explore the robustness of this model for water stressed crops. We established experiments to examine the combined effects of water stress, phenology, partitioning of biomass, and water-soluble carbohydrates (WSC), as driven by environment and variety, on the %N_c_ of wheat crops. We compared models where %N_c_ was plotted against biomass, growth stage and thermal time. The models were similarly scattered. Residuals of the %N_c_ - biomass model at anthesis were positively related to biomass, stem:biomass ratio, Δ^13^C and water supply, and negatively related to ear:biomass ratio and concentration of WSC. These are physiologically meaningful associations explaining the scatter of biomass-based dilution curves. Residuals of the thermal time model showed less consistent associations with these variables. The biomass dilution model developed for well-watered crops overestimates nitrogen deficiency of water-stressed crops, and a biomass-based model is conceptually more justified than developmental models. This has implications for diagnostic and modeling. As theory is lagging, a greater degree of empiricism might be useful to capture environmental, chiefly water, and genotype-dependent traits in the determination of critical nitrogen for diagnostic purposes. Sensitivity analysis would help to decide if scaling nitrogen dilution curves for crop water status, and genotype-dependent parameters are needed.

## Introduction

Nitrogen dilution curves relate a crop’s critical nitrogen concentration (%N_c_, the minimum nitrogen concentration required for maximum growth) to crop biomass (W). These curves are used in the diagnostics of crop nitrogen status and modeling, and have the form (Gastal et al., 2015):

(1) $$\%{\text{N}}_{\text{c}}\;\text{=}\;a{\text{W}}^{-b}$$

where, *b* is a dimensionless parameter that represents the nitrogen dilution relative to crop biomass and *a* is the crop nitrogen concentration when W = 1 t ha^-1^. The theoretical foundations of this model are strong (Greenwood et al., 1990; Lemaire and Gastal, 1997). The dilution of nitrogen occurs because at the start of the growing season, biomass mostly consists of leaves with high proportion of metabolic tissue and high nitrogen concentration, but as the crop grows, relatively more structural tissue, i.e., stem with a smaller nitrogen concentration, is produced. In addition, shading of older leaves causes nitrogen relocation within the plant. Concentration of nitrogen in biomass (%N) is

(2) $$\%\text{N}\;\text{=}\;(\%{\text{N}}_{\text{m}}{}^{\text{*}}{\text{W}}_{\text{m}}\text{+}\%{\text{N}}_{\text{s}}{}^{\text{*}}{\text{W}}_{\text{s}})\text{/W}$$

where subscripts *m* and *s* indicate metabolic and structural tissue, respectively.

The nitrogen nutrition index, defined as the ratio between actual and critical nitrogen concentration from dilution curves, is a robust measure of crop nitrogen status (Gastal et al., 2015). For crop management, the nitrogen nutrition index is used directly (Neuhaus et al., 2017) or as a reference to calibrate spectral indices (Colaço and Bramley, 2018; Cossani and Sadras, 2018). However, most experiments used to derive nitrogen dilution curves have been conducted in well-watered crops (e.g., Justes et al., 1994) and this may have contributed to the consistency of the parameters in eq. (1) (Gastal et al., 2015). In potato and tall fescue where dilution curves have been derived from crops exposed to contrasting water supply, %Nc declined with water stress (Bélanger et al., 2001; Errecart et al., 2014). Understanding the influence of crop water status on %Nc is important, as using dilution curves from well-watered crops may over-estimate nitrogen deficits in water-stressed crops (Sadras and Lemaire, 2014). In addition, this is theoretically interesting as it connects the water and nitrogen economies of the crop.

Variation in nitrogen dilution curves for wheat has been reported that relates to phenological development, hence the attempts to model critical nitrogen against phenological stage (Angus, 2007; Yue et al., 2012; Zhao et al., 2014; Ratjen and Kage, 2016). Variation in allometric relations, between organs as well as between structural and labile carbohydrates, partially underlies the effect of phenology on nitrogen dilution curves (Gastal et al., 2015; Hoogmoed and Sadras, 2016; Yan et al., 2016).

The aim of this study was to examine the combined effects of water stress, phenology, partitioning of biomass, and water-soluble carbohydrates (WSC) on the critical nitrogen concentrations of wheat crops.

## Materials and Methods

### Site Description

Field trials were conducted over three growing seasons (May – November, 2014–2016) in South Australia. In 2014, trials were established at Hart (33°45 ‘S, 138°24 ‘E) and Turretfield (34°32 S, 138°47 ‘E). In 2015 and 2016 trials were conducted at Roseworthy (34°31 ‘S, 138°57 ‘E). These sites have a Mediterranean climate with hot and dry summer, and wet and mild winter. Daily weather data were collected from nearby Australian Bureau of Meteorology’s weather stations^1^. Soils were sandy loam (Dermosol) at Hart, calcareous loam (Calcarosol) at Roseworthy and loam over clay (Chromosol) at Turretfield (Isbell, 1996).

### Trial Design

In 2014, we established a factorial experiment combining 4 varieties and 5 nitrogen rates in a randomized block design with 3 replicates. In 2015 and 2016, the variety by nitrogen factorial was grown under two water regimes: rainfed and irrigated. Crops were sown in the agronomically recommended window between 16 May and 30 June (Supplementary Table S1). Hereafter, “environment” refers to each of the six combinations of location, season, and water regimes.

We used four Australian cultivars: very early maturing *Axe* (Australian Grain Technologies), early maturity *Mace* (Australian Grain Technologies), and mid-long maturity *Scout* and *Trojan* (LongReach). Wheat was sown in plots of 10 m long, with six rows (25 cm spacing) and a sowing density of 210 plants m^-2^. Urea pellets were spread evenly in the plots by hand according to the following treatments: (1) 0 kg N ha^-1^; (2) 60 kg N ha^-1^ at seeding; (3) 120 kg N ha^-1^ split between seeding and tillering (GS20–23); (4) 180 kg N ha^-1^ split between seeding and tillering; (5) 240 kg N ha^-1^ split between seeding and tillering. At Hart in 2014, an additional treatment was included: (6) 180 kg N ha^-1^ split between seeding, tillering and stem elongation (GS31–32). In all cases, split applications were 50:50. In 2016 at Roseworthy, crops received 40 kg P ha^-1^ as superphosphate at sowing.

### Soil Moisture and Nitrogen at Sowing

Soils were sampled just before sowing with hand auger or hydraulic soil corer to 0.6 m deep and separated into 0.2 m soil layers to determine water and nitrogen content. For each soil layer, moisture was determined gravimetrically in 10 g subsamples. The remainder of the fresh soil was dried at 40°C for 1 week and crushed to pass a 2 mm mesh. KCl extractable NO_3_^-^-N and NH_4_^+^-N were measured in CSBP Soil and Plant Analysis Laboratory, Western Australia. Soil drying may have contributed to loss of N-NH_3_, which is a minor component of available nitrogen in our system.

### Phenology, Biomass and Nitrogen

Phenological stage was recorded regularly using Zadoks’ scale (Zadoks et al., 1974). Shoot biomass was sampled four to six times between GS23 and GS69. Shoots were cut close to the soil surface with a hand sickle, in two segments of 50 cm in the two central rows of the plot. Samples were oven dried at 60°C until constant weight and then weighed. A dry subsample was separated into leaves, stems, and ears when they were present. The plant components were weighed and ground separately using a mill (Thomas Wiley^®^ model 4, Swedesboro, NJ, United States) and analyzed for total nitrogen content by dry combustion (CSBP Soil and Plant Analysis Laboratory, Western Australia).

Stems from the anthesis samples were analyzed for WSC. Whole shoot samples of treatments that corresponded to %Nc were analyzed at anthesis for stable carbon isotope composition δ13C (‰). WSC and δ13C were analyzed by Environmental Analysis Laboratory, NSW, Australia. WSC was measured in a water extraction and then flow injection analysis using the alkaline ferricyanide decolouration method. δ13C was measured using a Thermo-Finnigan Delta V Plus Isotope Ratio Mass Spectrometer (IRMS). ^13^C discrimination (Δ^13^C) at anthesis was calculated as:

(3) $$\Delta^{\text{13}}\text{C}(‰)\;\text{=}\;(\delta_{\text{air}}\text{-}\delta_{\text{sample}})\text{/}(\text{1000+ }\delta_{\text{sample}})\;\times \;\text{1000}$$

in which δ_air_ is the ^13^C composition of air (-8 ‰, Farquhar et al., 1989) and δ_sample_ is the ^13^C composition of the sample. Smaller Δ^13^C means a higher degree of water stress (Errecart et al., 2014; Sadras et al., 2016).

### Statistical Analysis

Statistical analyses were performed in R (R Development Core Team, 2008). ANOVAs were performed using the *Anova()* function in the *car* package, and Tukey HSD *post hoc* tests used *HSD.test()* in the *agricolae* package. Model I, least square regression assuming only error in *y*, was performed using the *lm()* function. Model II, standardized major axes regression accounting for error in both *x* and *y* (Niklas, 1994) was performed using the *sma()* function in the *smatr* package. We used Model II where our aim was to derive parameters for allometric relations, as the parameters depend on the model (Niklas, 1994). Otherwise, when we only wanted to test for the magnitude of association, we used Model I as R^2^ is independent of the method.

### Critical Nitrogen Concentration

Shoot N concentration (%N_shoot_) was calculated using the relative weights of the plant components and their nitrogen concentrations:

(4) $$\%{\text{N}}_{\text{shoot}}\;\text{=}\;({\text{W}}_{\text{stem}}{}^{\text{*}}\%{\text{N}}_{\text{stem}}{\text{+W}}_{\text{leaf}}{}^{\text{*}}\%{\text{N}}_{\text{leaf}}{\text{+W}}_{\text{ear}}{}^{\text{*}}\%{\text{N}}_{\text{ear}})\text{/}({\text{W}}_{\text{stem}}{\text{+W}}_{\text{leaf}}{\text{+W}}_{\text{ear}})$$

where W is biomass, and %N the nitrogen concentration of the plant component. The %Nc at each sampling date was calculated for each environment and each variety following the method of Greenwood et al. (1990). For each sampling time and variety, biomass dry weight was compared among the nitrogen treatments using one-way-ANOVA. If differences among the nitrogen treatments were not significant, the data from that sampling date and variety were not used. This lack of nitrogen effect on biomass was verified in 42 out of 120 samples (30 sampling times × 4 varieties), and was likely related to high nitrogen available in soil at sowing in some trials (Supplementary Table S1). If differences were significant (*P* < 0.1), Duncan’s *post hoc* test was performed following Marino et al. (2004) to reduce the chance of type II error. The treatment with the highest mean biomass was identified. If more than one treatment resulted in similarly high biomass i.e., not significantly different (*P* > 0.1), the nitrogen treatment resulting in the lowest shoot nitrogen concentration was selected with corresponding %Nc and biomass.

Three models were used to describe nitrogen dilution: (1) the classical nitrogen dilution curve, that relates %Nc to biomass (*biomass model* hereafter); (2) a dilution curve that relates %Nc to thermal time from sowing with base temperature = 0°C (*thermal time model*); and (3) a categorical model where %Nc is related to growth stages (*growth stage model*). For the first two models, %N_c_ = *a* X^-^*^b^* curves were fit using standardized major axis regression, where X is either biomass (t ha^-1^) or thermal time (°C d). No curve was fitted for the *growth stage model* as the numerical scale neither has a true biological meaning nor is quantitative; instead, average %Nc and standard deviations were calculated for the data pooled in five growth stage intervals: 30–34, 35–39, 40–49, 50–59, and 60–69.

### Residual Analysis

Residual analysis was used to explore drivers of scatter around the *biomass* and *thermal time* models. Residuals were calculated as the difference between the actual %Nc and the fitted curves. ANOVA was used to test for the effects of variety and environment on the residuals (Sadras and Moran, 2012; Badyaev et al., 2017). Model II regression was used to test for associations between the residuals of each nitrogen dilution model and the following variables (1) biomass, (2) mass fractions of leaf, stem and ear, (3) WSC concentration in stem and shoot, (4) Δ^13^C, (5) water supply calculated as available soil water at sowing plus rainfall and irrigation, (6) reference evapotranspiration ETo (Allen et al., 1998), and (7) water supply per unit ETo.

## Results

### Growing Conditions

**FIGURE 1:**
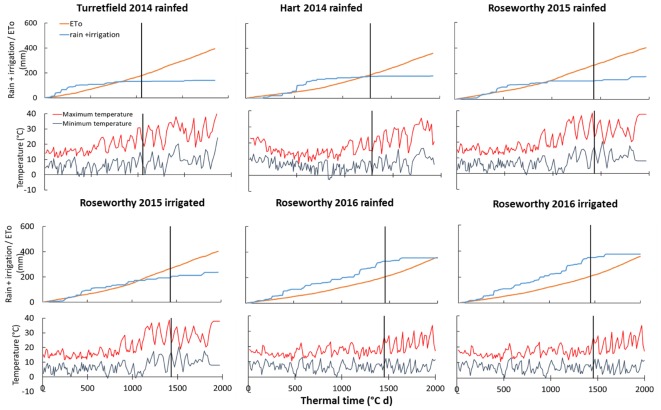
Cumulative reference evapotranspiration (ETo), cumulative rain and irrigation, and temperature against thermal time from sowing in six environments in South Australia. Vertical lines indicate anthesis sampling. ETo is calculated with the FAO-56 method (Allen et al., 1998).

Mineral soil nitrogen at sowing ranged from 34 to 345 kg ha^-1^ (Supplementary Table S1). Crop available water at sowing ranged from negligible to 83 mm in the top 0.6 m soil layer (Supplementary Table S1); dry soils at sowing are a common feature of these environments with dominant winter rainfall and unlikely rain during fallow (Sadras and Rodriguez, 2007). The 2014–2016 growing seasons differed in amount and distribution of precipitation, and in temperature (**Figure 1**). In 2015, seasonal precipitation started relatively late, and a hot and dry finish caused water deficit even in irrigated crops. In contrast, precipitation was above average and irrigation was applied only a few times early in 2016. Intensity of water deficit was further quantified with Δ^13^C and water budget in section *Sources of scatter in the* biomass *model* below.

### Dynamics of Growth and Nitrogen Uptake

**FIGURE 2:**
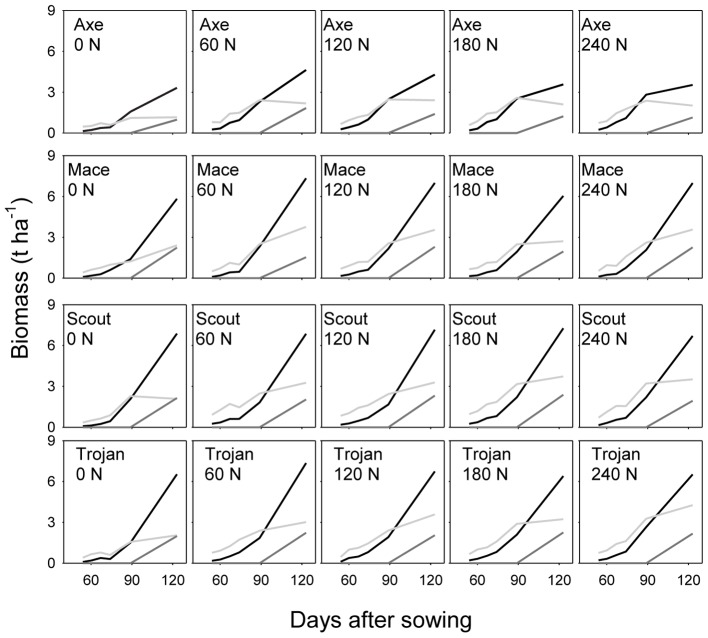
Dynamics of dry matter in leaf (light gray), stem (black), and ear (dark gray) for wheat crops in a factorial experiment combining four varieties and five rates of nitrogen fertilizer. Data from irrigated crops at Roseworthy, 2016.

**FIGURE 3:**
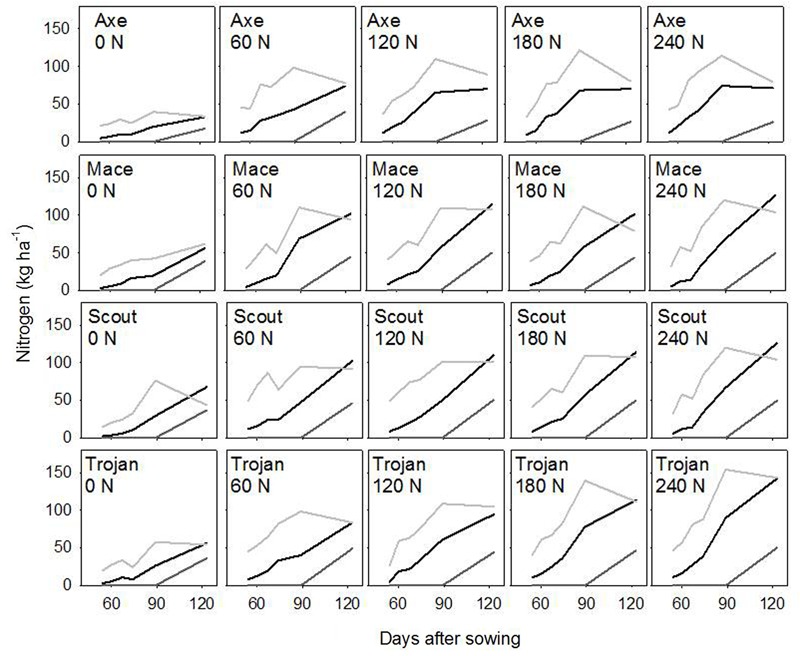
Dynamics of nitrogen uptake in leaf (light gray), stem (black), and ear (dark gray) for wheat crops in a factorial experiment combining four varieties and five rates of nitrogen fertilizer. Data from irrigated crops at Roseworthy, 2016.

Across environments, nitrogen rate affected biomass of stem, leaf and ear (all *P* < 0.0001), and amount of nitrogen in each of these organs (all *P* < 0.0001). Variety affected leaf biomass (*P* < 0.0001), amount of nitrogen in stem (*P* < 0.05), and amount of nitrogen in leaf (*P* < 0.001). **Figure 2** illustrates the effect of nitrogen rate and variety for biomass, and **Figure 3** for nitrogen uptake, highlighting the larger effect of nitrogen rate on nitrogen uptake compared to biomass conducive to changes in nitrogen concentration, which are analyzed in the following sections.

### Biomass Model

**FIGURE 4:**
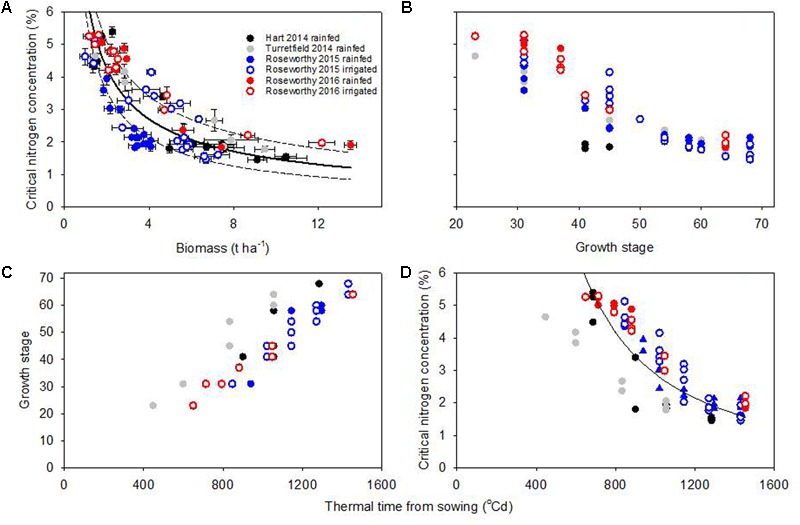
**(A)** Wheat critical nitrogen concentration vs. biomass, where the fitted model (solid line) is %Nc = 6.75 ^∗^ W^-0.66^ (R^2^= 0.77, *P* < 0.0001) and the 95% confidence intervals (dotted lines) are %Nc = 7.61 ^∗^ W^-0.58^ (upper) and %Nc = 5.99 ^∗^ W^-0.75^ (lower). **(B)** Critical nitrogen concentration vs. Zadoks’ growth stage. **(C)** Relationship between growth stage and thermal time from sowing. **(D)** Critical nitrogen concentration vs. thermal time from sowing, where the fitted model is %N_c_ = 2.4^∗^10^5∗^thermal time^-1.64^ (R^2^ = 0.82, *P* < 0.0001); the scatter of data did not allow to fit confidence intervals.

Fitting eq. (1) to our data returned a R^2^ = 0.77 (*P* < 0.0001), and showed substantial scatter with small standard errors in %Nc (**Figure 4A**). Across growing conditions, varieties, and crop stages, the median coefficient of variation of %Nc was 5.5%. Further, the coefficient of variation of %Nc was similar in the rainfed and irrigated experiments at Roseworthy in both seasons: 4.5% under irrigation vs. 4.0% under rainfed conditions in 2015, and 4.2% under irrigation vs. 4.0% under rainfed conditions in 2016. These relatively small errors together with the clustering of some experiments in **Figure 4A** indicate that crop and environmental factors were more likely sources of scatter. For example, the %Nc under severe water deficit in rainfed crops at Roseworthy 2015 (**Figure 1**) clustered below the dilution curve (filled blue circles in **Figure 4A**).

### Growth Stage and Thermal Time Models

To analyze the impact of phenology on dilution curves we plotted %N_c_ against growth stage; the scatter was large at early stages, and seemed to diminish toward flowering (**Figure 4B**). **Table 1** summarizes %N_c_ as average and standard deviation for selected developmental windows. As development related with thermal time (**Figure 4C**), we plotted %N_c_ against thermal time from sowing (**Figure 4B**). The R^2^ of 0.82 for the *thermal time model* was slightly better than that for the *biomass model*, but substantial scatter remained.

**Table 1:** Average (± standard deviation) critical nitrogen concentration of wheat for Zadoks’ growth stages from early stem elongation to anthesis (Zadoks et al., 1974).

Growth stage	Critical *N* (%)
30–34	4.7 ± 0.5
35–39	4.4 ± 0.2
40–49	2.9 ± 0.7
50–59	2.1 ± 0.3
60–69	1.8 ± 0.2

### Comparison of Scatter in *Biomass* and *Thermal Time* Models

**FIGURE 5:**
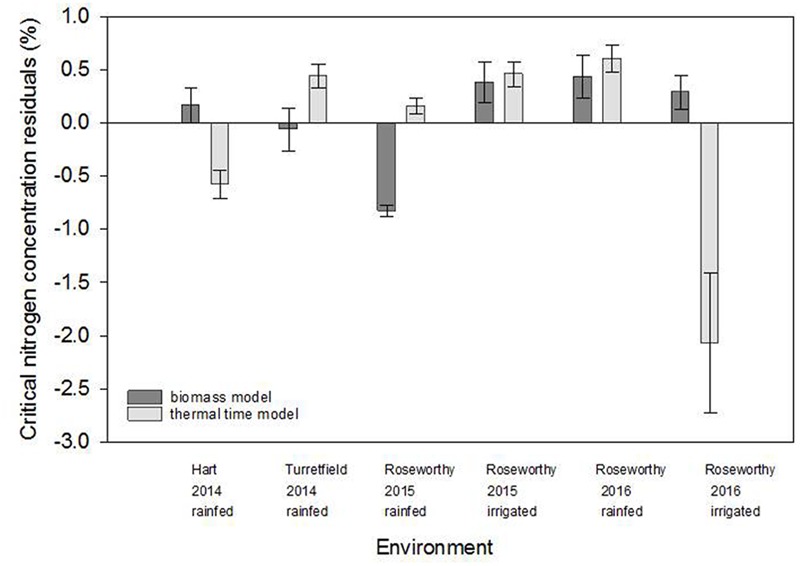
Comparison of residuals for the *biomass* and *thermal time* models in five environments resulting from combination of locations, seasons and water regimes. Values are means ± standard error.

**Figure 5** compares the residuals for the *biomass* and *thermal time* models. Positive residuals indicate under-estimation and negative residuals an over-estimation of the actual %N_c_ relative to the fitted model. For both models, ANOVA of residuals revealed significant environmental effect (*P* < 0.0001), and lack of variety (*P* > 0.66) and variety-by-environment interaction (*P* > 0.81). For the *biomass model*, residuals were positive and moderate in most environments, except for rainfed crops at Roseworthy 2015, where residuals were strongly negative. For the *thermal time* model, the residuals were particularly large for rainfed crops at Turretfield 2014.

### Sources of Scatter in the *Biomass Model*

**FIGURE 6:**
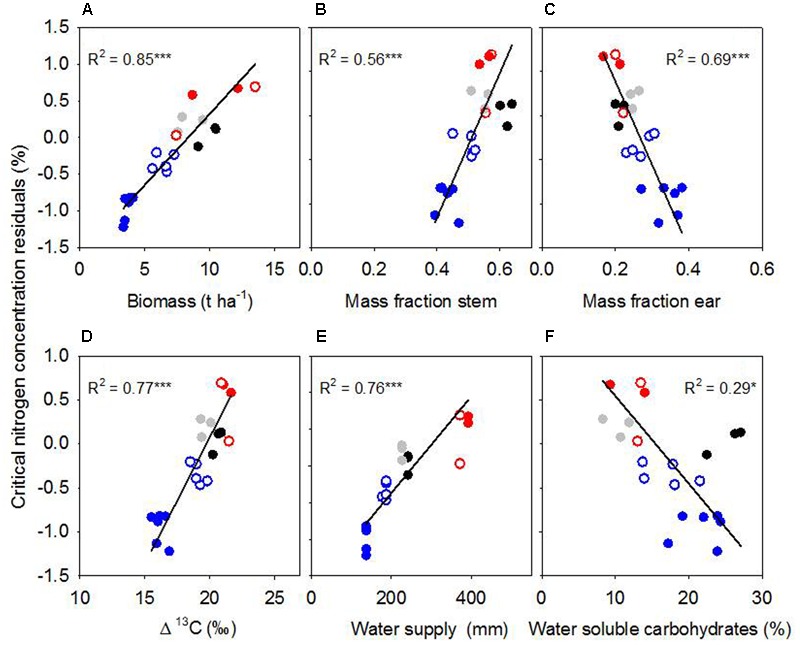
Residuals from the critical nitrogen concentration vs. biomass model at anthesis in relation to **(A)** biomass, **(B)** mass fraction stem, **(C)** mass fraction ear, **(D)** Δ^13^, **(E)** water supply, and **(F)** water-soluble carbohydrates in stem. Lines are Model II regressions, with ^∗∗∗^*P* < 0.001, ^∗^*P* < 0.05. Symbols: Hart 2014 rainfed (black); Turretfield 2014 rainfed (gray); Roseworthy 2015 rainfed (filled blue); Roseworthy 2015 irrigated (open blue); Roseworthy 2016 rainfed (filled red); Roseworthy 2016 irrigated (open red).

To further explore the sources of scatter in %Nc among the environments, we compared total biomass, and allocation of biomass to leaf, stem and ear. Maximum biomass at anthesis ranged from 3.7 t ha^-1^ for rainfed crops at Roseworthy 2015 to 10.5 t ha^-1^ for irrigated crops at Roseworthy 2016 (*P* < 0.01). Much of this variation was related to water, as biomass at anthesis related closely with both water supply and Δ^13^C (**Table 2**). The mass fractions of leaf, stem and ear at similar growth stages also differed among the environments and correlated with total biomass at GS31 and GS60–69 (**Table 3**). The mass fraction of stem correlated positively with total biomass, while mass fraction of leaf correlated negatively, except at anthesis where there was no correlation with leaf, and instead the mass fraction ear correlated negatively with total biomass. Residuals of critical nitrogen concentration at anthesis correlated positively with both biomass and mass fraction stem, and negatively with mass fraction ear (**Figures 6A–C**).

**Table 2:** Correlation matrix of crop traits at anthesis (biomass, mass stem fraction, mass ear fraction, Δ^13^C, concentration of water soluble carbohydrates) and environmental factor (water supply) that contributed to the scatter of the %Nc-biomass curve.

	Biomass	Mass fraction stem	Mass fraction ear	Δ^13^C	Water supply
Mass fraction stem	0.89^∗∗^				
Mass fraction ear	-0.86^∗∗^	-0.90^∗∗^			
Δ^13^C	0.92^∗∗^	0.88^∗∗^	-0.88^∗∗^		
Water supply	0.87^∗∗^	0.65^∗∗^	-0.81^∗∗^	0.90^∗∗^	
Water soluble carbohydrates	-0.41	-0.10	0.28	-0.39	-0.50^∗^

*^∗∗^*P* < 0.01; ^∗^*P* ≤ 0.05.*

**Table 3:** Correlation coefficient (r) for leaf, stem and ear mass fraction with total biomass and Δ^13^C among the six environments.

Variable	Growth stage	*N*	Total biomass	Δ^13^C
Mass fraction stem	23–69	78	0.81**	-0.40**
	31	17	0.48*	-0.56*
	40–49	19	0.36	-0.16
	60–69	21	0.88**	0.88**
Mass fraction leaf	23–69	78	-0.81**	0.65**
	31	17	-0.54*	0.59*
	40–49	19	-0.42	0.07
	60–69	21	-0.29	-0.23
Mass fraction ear	60–69	21	-0.86**	0.88**

*^∗^*P* ≤ 0.05, ^∗∗^*P* < 0.01.*

Crop water stress varied among the environments as indicated by the range of Δ^13^C from 21‰ for both irrigated and rainfed crops at Roseworthy 2016, to 15.9 ‰ in the most stressed crops at Roseworthy 2015 (*P* < 0.01) (**Figure 6D**). Water supply and Δ^13^C correlated, reinforcing the confidence in our coarse water budget (**Table 2**). Residuals of %Nc from the *biomass* model correlated positively with Δ^13^C and water supply and negatively with reference evapotranspiration (**Figures 6D,E** and **Table 4**).

**Table 4:** Correlation coefficients (r) between residuals from *biomass* and *thermal time* nitrogen dilution models, and shoot biomass, mass fraction of stem, leaf, ear, concentration of water-soluble carbohydrates (WSC) in stem and shoot, Δ^13^C, water supply, cummulative reference evapotranspiration (ETo), and cummulative water supply per unit reference evapotranspiration.

Variable	Model	
	Biomass	Thermal time
Biomass^1^	0.92**	-0.23
Mass fraction stem^1^	0.75**	-0.47*
Mass fraction leaf^1^	-0.07	0.57**
Mass fraction ear^1^	-0.83**	0.21
Δ^13^C^1^	0.88**	-0.25
WSC in stem^1^	-0.54*	0.04
WSC in shoot^1^	-0.17	-0.18
Water supply^2^	0.87**	0.16
ETo^2^	-0.68**	0.61**
Water supply/ ETo^2^	0.91**	-0.16

*Water supply was calculated as available water in the soil at sowing plus irrigation and rainfall. ^∗^*P* ≤ 0.05, ^∗∗^*P* < 0.01. ^*1*^Measured at anthesis; ^*2*^Cumulative from sowing to anthesis.*

Average WSC concentration in stem at anthesis differed among the environments, from 10.3% at Turretfield to 25.1% at Hart, both rainfed in 2014. Residuals from the *biomass* model correlated negatively with WSC (**Figure 6F**). WSC increased under water deficit as indicated by the relationship between WSC at anthesis and water supply (**Table 2**); the relationship between WSC and Δ^13^C was negative but did not reach significance (*P* = 0.08).

### Sources of Scatter in the *Thermal Time* Model

With few exceptions, residuals for the *thermal time* model were weakly associated with the explanatory variables that correlated with the residuals from the *biomass* model (**Table 4**). For example, residuals from the *thermal time* model did not correlate with biomass, and correlations were weaker, and of opposite sign than those from the *biomass* model for stem mass fraction and ear mass fraction (**Table 4**). The consistent associations between residuals and water-related variables found for the *biomass* model were absent for the *thermal time* model, except for reference evapotranspiration (**Table 4**).

## Discussion

We examined the combined effects of water stress, phenology, partitioning of biomass among organs, and partitioning between structural and WSC on the critical nitrogen concentration of wheat crops. Tight nitrogen dilution curves are often derived for well-watered crops, e.g., wheat (Justes et al., 1994) and maize (Plénet and Lemaire, 1999); larger scatter was observed in potato crops under variable water supply (Bélanger et al., 2001). Here we found scattered dilution curves for crops grown under varying water deficits (**Figure 4A**). We tested three nitrogen dilution models and, informed by physiological principles, used analysis of residuals to explore the sources of scatter in the *biomass* and *thermal time* models (**Figures 5, 6** and **Table 4**). Small errors in %Nc (**Figure 4A**) combined with analysis of residuals supported the conclusion that the large scatter in biomass-based dilution curves was caused by physiologically meaningful drivers, particularly water supply and patterns of dry matter allocation (**Figure 6**).

### Phenological Development

Phenology-dependent changes in allocation of biomass between metabolic and structural tissue (eq. 2) explain part of the scatter in the *biomass* model. Zhao et al. (2014) used a *growth stage* model to account for phenological development, but fitting curves to growth stages is not justified because the independent variable is nominal rather than quantitative. Instead, we analyzed critical nitrogen concentration against growth stage in a discrete model (**Figure 4B** and **Table 1**), and used a thermal time scale to fit a quasi-developmental model (**Figures 4C,D**) and analyze residuals (**Figure 5** and **Table 4**). We still found a large spread of %Nc within growth stages, especially early in the season, and statistically similar scatter in the *thermal time* and *biomass* models (**Figure 5**). In the earlier growth stages (stem elongation to booting), the leaf : stem ratios changed more rapidly than in later growth stages (ear emergence and anthesis). Accuracy in determining phenology is important, for example any comparison between early (GS31) and late (GS37) stem elongation will incur a large effect on leaf : stem ratio and thus %Nc. Our average %Nc between GS31 and GS49 was higher than reported for winter wheat by Zhao et al. (2014) (and other references therein) but similar at anthesis (GS60–69).

### Partitioning of Biomass

Beyond phenological stages, genetic and environmental factors affect partitioning of biomass with implications for nitrogen-biomass allometry (Niklas, 2004; Weiner, 2004; Poorter et al., 2012). Within growth stages, there was still a difference in mass fractions of leaf, stem and ear among the environments. Total biomass correlated positively with stem mass fraction and negatively with leaf mass fraction. Water stress affects wheat allometry (Kumakov et al., 2001; Ratjen et al., 2016). In our dataset, the mass fractions of stem and ear correlated best with Δ^13^C at anthesis, with a decrease in stem, and an increase in ear mass fraction with increasing water stress, but no change in leaf mass fraction. Kumakov et al. (2001) found a similar result in wheat, and Poorter et al. (2012) found the same trend in a meta-analysis of intra-specific variation and environmental control of biomass partitioning. In contrast, Ratjen et al. (2016) found an increase in stem growth rate relative to leaf growth rate under water stress during stem elongation. Importantly, allocation of biomass to ear and stem, but not to leaf, explained a significant proportion of the scatter in the *biomass* model (**Figures 6B,C** and **Table 4**). Allocation of biomass to leaf was, however, a factor accounting for some of the scatter in the *thermal time* model (**Table 4**), highlighting again the interplay between phenology and biomass partitioning.

### Water Stress and Water-Soluble Carbohydrates

As expected from theory (Sadras and Lemaire, 2014; Hoogmoed and Sadras, 2016) and empirical evidence in other species (Bélanger et al., 2001; Errecart et al., 2014), we found that the critical nitrogen concentration of wheat was lower under water stress, and with high concentration of WSC (**Figure 6**). Three independent water-related variables associated with the residuals of the *biomass model*: Δ^13^C, water supply and reference evapotranspiration (**Figure 6** and **Table 4**). Further, the strong correlation between biomass and Δ^13^C (**Table 2**) indicates the association between residuals and biomass (**Figure 6A**) was likely mediated by the effect of water deficit on biomass. Our dilution curve thus returned lower critical nitrogen compared with the original curve for well-watered wheat in France (Justes et al., 1994). Dilution curves for winter wheat in China (Yin et al., 2018) and for spring wheat in Canada (Ziadi et al., 2010) were also below the original curve for well-watered crops, and this is partially attributable to water deficit.

Under our experimental conditions, concentration of WSC increased under water deficit. Previous studies showed that stress decreased (Foulkes et al., 2002, 2007; Ehdaie et al., 2006; Rebetzke et al., 2008) or increased WSC (Zhu et al., 2009; Saint Pierre et al., 2010). In our study, the association between residuals of the *biomass model* and water supply was partially mediated by the effects of water supply on WSC. Our experiment confirmed our early prediction of smaller critical nitrogen concentration in crops where genotype, environment and their interaction favor high storage of labile carbohydrates (Hoogmoed and Sadras, 2016).

### Statistical, Agronomic and Modeling Implications

Studies of nitrogen dilution curves rarely specify the method used to fit curves; but whether regressions are fitted with Model I (least squares) or Model II (standardized maximum axis), is unimportant in close-fitting relationships as parameters are similar if R^2^ is high (Niklas, 1994), which is usually the case (Justes et al., 1994; Plénet and Lemaire, 1999). Where the growing conditions scatter dilution curves, as in this study, Model II should be used (Niklas, 1994) and reporting confidence intervals of parameters would be useful for comparisons (**Figure 4A**, see also Zhao et al., 2014).

The water-driven scatter in nitrogen dilution curves has agronomic and modeling implications. In both cases, sensitivity analysis would help to decide if adjustments to capture the effect of water deficit are necessary. In Mediterranean-type environments, unreliable seasonal rainfall is a large source of uncertainty that favors a conservative approach to nitrogen fertilization in risk-averse farmers (Monjardino et al., 2013). This uncertainty in rainfall and yield provides an agronomic background to evaluate the errors in estimating crop nitrogen status using alternative dilution curves. In this context, locally estimated critical nitrogen for particular phenostages could be useful.

## Conclusion

The %Nc -biomass dilution curve developed for well-watered crops would overestimate nitrogen deficiency of water-stressed wheat. The effects of water deficit on critical nitrogen concentration at anthesis are likely mediated by two changes in allocation of carbon: between stem and ear, and between structural and labile, as indicated by the increase in WSC with stress. The causal connections between water stress and these two aspects of carbon allocation deserve refined investigation. Biomass-based models are conceptually superior to developmental-based models, as both showed statistically similar scatter but the former has a stronger theoretical foundation, reinforced by the close relationships between model residuals and physiologically meaningful factors. Conceptually, nitrogen-biomass dilution curves need to account for genotypic and environmental sources of variation in biomass allocation, including phenology and WSC.

## Author Contributions

MH carried out the experiments, analyzed and interpreted the data, and wrote the manuscript. VS designed the study, analyzed and interpreted the data, and wrote the manuscript.

## Conflict of Interest Statement

The authors declare that the research was conducted in the absence of any commercial or financial relationships that could be construed as a potential conflict of interest.

## Footnotes

1 https://www.longpaddock.qld.gov.au/silo/index.html

## References

[B1] Allen R. G. Pereira L. S. Raes D. Smith M. (1998). *Crop Evapotranspiration: Guidelines for Computing Crop Water Requirements.* Rome: FAO.

[B2] Angus J. F. (2007). “Should nitrogen dilution curves be expressed in relation to biomass or development?,” in *Towards a Better Efficiency in N Use* eds Bosch A. Teira M. R. Vilar J. M. (Lleida: Editorial Milenio) 305–308.

[B3] Badyaev A. V. Potticary A. L. Morrison E. S. (2017). Most colorful example of genetic assimilation? exploring the evolutionary destiny of recurrent phenotypic accommodation. *Am. Nat.* 190 266–280. 10.1086/692327 28731798

[B4] Bélanger G. Walsh J. R. Richards J. E. Milburn P. H. Ziadi N. (2001). Critical nitrogen curve and nitrogen nutrition index for potato in eastern Canada. *Am. J. Potato Res.* 78 355–364. 10.1007/BF02884344

[B5] Colaço A. F. Bramley R. G. V. (2018). Do crop sensors promote improved nitrogen management in grain crops? *Field Crop Res.* 218 126–140. 10.1016/j.fcr.2018.01.007

[B6] Cossani C. M. Sadras V. O. (2018). Water-nitrogen co-limitation in grain crops. *Adv. Agron.* 150 (in press). Available at: https://www.elsevier.com/books/advances-in-agronomy/sparks/978-0-12-815175-4

[B7] Ehdaie B. Alloush G. A. Madore M. A. Waines J. G. (2006). Genotypic variation for stem reserves and mobilization in wheat: II. Postanthesis changes in internode water-soluble carbohydrates. *Crop Sci.* 46 2093–2103. 10.2135/cropsci2006.01.0013

[B8] Errecart P. M. Agnusdei M. G. Lattanzi F. A. Marino M. A. Berone G. D. (2014). Critical nitrogen concentration declines with soil water availability in tall fescue. *Crop Sci.* 54 318–330. 10.2135/cropsci2013.08.0561

[B9] Farquhar G. D. Ehleringer J. R. Hubick K. T. (1989). Carbon isotope discrimination and photosynthesis. *Annu. Rev. Plant Physiol. Plant Mol. Biol.* 40 503–537. 10.1146/annurev.pp.40.060189.002443

[B10] Foulkes M. J. Scott R. K. Sylvester-Bradley R. (2002). The ability of wheat cultivars to withstand drought in UK conditions: formation of grain yield. *J. Agric. Sci.* 138 153–169. 10.1017/S0021859601001836

[B11] Foulkes M. J. Sylvester-Bradley R. Weightman R. Snape J. W. (2007). Identifying physiological traits associated with improved drought resistance in winter wheat. *Field Crop Res.* 103 11–24. 10.1016/j.fcr.2007.04.007

[B12] Gastal F. Lemaire G. Durand J. L. Louarn G. (2015). “Quantifying crop responses to nitrogen and avenues to improve nitrogen-use efficiency,” in *Crop Physiology: Applications for Genetic Improvement and Agronomy* eds Sadras V. O. Calderini D. F. (San Diego, CA: Academic Press) 161–206.

[B13] Greenwood D. J. Lemaire G. Gosse G. Cruz P. Draycott A. Neeteson J. J. (1990). Decline in percentage N of C3 and C4 crops with increasing plant mass. *Ann. Bot.* 66 425–436. 10.1093/oxfordjournals.aob.a088044

[B14] Hoogmoed M. Sadras V. O. (2016). The importance of water-soluble carbohydrates in the theoretical framework for nitrogen dilution in shoot biomass of wheat. *Field Crop Res.* 193 196–200. 10.1016/j.fcr.2016.04.009

[B15] Isbell R. F. (1996). *The Australian Soil Classification.* Melbourne, VIC: CSIRO Publishing.

[B16] Justes E. Mary B. Meynard J. M. Machet J. M. Thelierhuche L. (1994). Determination of a critical nitrogen dilution curve for winter wheat crops. *Ann. Bot.* 74 397–407. 28928757

[B17] Kumakov V. A. Evdokimova O. A. Buyanova M. A. (2001). Dry matter partitioning between plant organs in wheat cultivars differing in productivity and drought resistance. *Russ. J. Plant Physiol.* 48 359–363. 10.1023/A:1016670501685

[B18] Lemaire G. Gastal F. (1997). “N uptake and distribution in plant canopies,” in *Diagnosis of the Nitrogen Status in Crops* ed. Lemaire G. (Berlin: Springer-Verlag) 3–41. 10.1007/978-3-642-60684-7_1

[B19] Marino M. A. Mazzanti A. Assuero S. G. Gastal F. Echeverria H. E. Andrade F. (2004). Nitrogen dilution curves and nitrogen use efficiency during winter-spring growth of annual ryegrass. *Agron. J.* 96 601–607. 10.2134/agronj2004.0601

[B20] Monjardino M. Mcbeath T. M. Brennan L. Llewellyn R. S. (2013). Are farmers in low-rainfall cropping regions under-fertilising with nitrogen? A risk analysis. *Agric. Syst.* 116 37–51. 10.1016/j.agsy.2012.12.007

[B21] Neuhaus A. Hoogmoed M. Sadras V. (2017). Closing the yield gap for wheat and canola through an adjusted nitrogen nutrition index. *Better Crops Plant Food* 101 16–18.

[B22] Niklas K. J. (1994). *Plant Allometry: The Scaling of Form and Process.* Chicago, IL: University of Chicago Press.

[B23] Niklas K. J. (2004). Plant allometry: is there a grand unifying theory? *Biol. Rev.* 79 871–889. 10.1017/S1464793104006499 15682874

[B24] Plénet D. Lemaire G. (1999). Relationships between dynamics of nitrogen uptake and dry matter accumulation in maize crops. Determination of critical N concentration. *Plant Soil* 216 65–82. 10.1023/A:1004783431055

[B25] Poorter H. Niklas K. J. Reich P. B. Oleksyn J. Poot P. Mommer L. (2012). Biomass allocation to leaves, stems and roots: meta-analyses of interspecific variation and environmental control. *New Phytol.* 193 30–50. 10.1111/j.1469-8137.2011.03952.x 22085245

[B26] R Development Core Team (2008). *R: A Language and Environment for Statistical Computing.* Vienna: R Foundation for Statistical Computing.

[B27] Ratjen A. M. Kage H. (2016). Nitrogen-limited light use efficiency in wheat crop simulators: comparing three model approaches. *J. Agric. Sci.* 154 1090–1101. 10.1017/S0021859615001082

[B28] Ratjen A. M. Neukam D. Kage H. (2016). A simple drought-sensitive model for leaf:stem partitioning of wheat. *J. Agron. Crop Sci.* 202 300–308. 10.1111/jac.12165

[B29] Rebetzke G. J. Van Herwaarden A. F. Jenkins C. Weiss M. Lewis D. Ruuska S. (2008). Quantitative trait loci for water-soluble carbohydrates and associations with agronomic traits in wheat. *Aust. J. Agric. Res.* 59 891–905. 10.1071/AR08067

[B30] Sadras V. O. Lake L. Li Y. Farquharson E. A. Sutton T. (2016). Phenotypic plasticity and its genetic regulation for yield, nitrogen fixation and δ13C in chickpea crops under varying water regimes. *J. Exp. Bot.* 67 4339–4351. 10.1093/jxb/erw221 27296246

[B31] Sadras V. O. Lemaire G. (2014). Quantifying crop nitrogen status for comparisons of agronomic practices and genotypes. *Field Crop Res.* 164 54–64. 10.1016/j.fcr.2014.05.006

[B32] Sadras V. O. Moran M. A. (2012). Elevated temperature decouples anthocyanins and sugars in berries of Shiraz and Cabernet Franc. *Aust. J. Grape Wine Res.* 18 115–122. 10.1111/j.1755-0238.2012.00180.x

[B33] Sadras V. O. Rodriguez D. (2007). The limit to wheat water use efficiency in eastern Australia. II. Influence of rainfall patterns. *Aust. J. Agric. Res.* 58 657–669. 10.1071/AR06376

[B34] Saint Pierre C. Trethowan R. Reynolds M. (2010). Stem solidness and its relationship to water-soluble carbohydrates: association with wheat yield under water deficit. *Funct. Plant Biol.* 37 166–174. 10.1071/FP09174

[B35] Weiner J. (2004). Allocation, plasticity and allometry in plants. *Perspect. Plant Ecol. Evol. Syst.* 6 207–215. 10.1078/1433-8319-00083

[B36] Yan B. Ji Z. Fan B. Wang X. He G. Shi L. (2016). Plants adapted to nutrient limitation allocate less biomass into stems in an arid-hot grassland. *New Phytol.* 211 1232–1240. 10.1111/nph.13970 27101947

[B37] Yin M. Li Y. Xu L. Shen S. Faang H. (2018). Nutrition diagnosis in winter wheat based on critical nitrogen dilution curves. *Crop Sci.* 58 1–10. 10.2135/cropsci2017.05.0326

[B38] Yue S. C. Meng Q. F. Zhao R. F. Li F. Chen X. P. Zhang F. S. (2012). Critical nitrogen dilution curve for optimizing nitrogen management of winter wheat production in the North China plain. *Agron. J.* 104 523–529. 10.2134/agronj2011.0258

[B39] Zadoks J. C. Chang T. T. Konzak C. F. (1974). A decimal code for the growth stages of cereals. *Weed Res.* 14 415–421. 10.1111/j.1365-3180.1974.tb01084.x

[B40] Zhao Z. Wang E. Wang Z. Zang H. Liu Y. Angus J. F. (2014). A reappraisal of the critical nitrogen concentration of wheat and its implications on crop modeling. *Field Crop Res.* 164 65–73. 10.1016/j.fcr.2014.05.004

[B41] Zhu L. Liang Z. S. Xu X. Li S. H. Monneveux P. (2009). Evidences for the association between carbon isotope discrimination and grain yield-Ash content and stem carbohydrate in spring wheat grown in Ningxia (Northwest China). *Plant Sci.* 176 758–767. 10.1016/j.plantsci.2009.02.018

[B42] Ziadi N. Belanger G. Claessens A. Lefebvre L. Cambouris A. N. Tremblay N. (2010). Determination of a critical nitrogen dilution curve for spring wheat. *Agron. J.* 102 241–250. 10.2134/agronj2009.0266

