# Supplementary material for: Water Stress Scatters Nitrogen Dilution Curves in Wheat
Source: Front Plant Sci. 2018 Apr 6;9:406. doi: 10.3389/fpls.2018.00406 (PMC5897705; doi:10.3389/fpls.2018.00406)
Supplement: Supplementary file 1 [file Table_1.DOCX]

Supplementary Material

**Nitrogen Dilution Curves of Wheat are Less Robust than Usually Assumed: Influence of Crop Water Status, Phenology, Biomass Partitioning, and Water-Soluble Carbohydrates**

Marianne Hoogmoed, Victor O Sadras*

*South Australian Research and Development Institute, Waite Campus, Australia*

*** Correspondence:** Victor Sadras, South Australian R&D Institute

victor.sadras@sa.gov.au

Table S1. Trial details, and crop available soil water and mineral N at sowing (0-0.6 m depth).

| **Location** | **Year** | **Water treatment** | **Sowing date** | **Harvest date** | **Available soil water content (mm)^b^** | **Total mineral N (kg ha^-1^)** |
| --- | --- | --- | --- | --- | --- | --- |
| Hart | 2014 | Rainfed | 16/05/14 | 28/10/14 | 29 | 95 |
| Turretfield | 2014 | Rainfed | 30/06/14 | 13/11/14 | 83 | 345 |
| Roseworthy | 2015 | Rainfed | 19/06/15 | 12/11/15 | ≈ 0 | 34 |
| Roseworthy | 2015 | Irrigated | 19/06/15 | 23/11/15 | ≈ 0 | 34 |
| Roseworthy | 2016^a^ | Rainfed | 02/06/16 | 06/12/16 | 41 | 184 (0 N plots)  375 (240 N plots) |
| Roseworthy | 2016^a^ | Irrigated | 02/06/16 | 06/12/16 | 43 | 140 (0 N plots)  281 (240 N plots) |

^a^ In 2016, the same treatments were allocated to the previous year’s plots, to avoid high residual N, when for example a 0 N treatment would be located on top of the previous year’s 240 N treatment.

^b^ Derived from actual gravimetric measurements of soil water, and lower limits from the APSIM soil data base <https://www.apsim.info/Products/APSoil.aspx>.
